# Supplementary material for: Packham’s Triumph Pears (Pyrus communis L.) Post-Harvest Treatment during Cold Storage Based on Chitosan and Rue Essential Oil
Source: Molecules. 2021 Jan 30;26(3):725. doi: 10.3390/molecules26030725 (PMC7866551; doi:10.3390/molecules26030725)
Supplement: Supplementary file 1 [file molecules-26-00725-s001.pdf]

Supplementary Information

Packham's Triumph Pears (*Pyrus communis* L.) Post-Harvest Treatment During Cold Storage Based on Chitosan and Rue Essential oil

Yeimmy Peralta-Ruiz [1,2], Carlos David Grande-Tovar [3,*], Diana Paola Navia Porras [4], Angie Sinning-Mangonez [2,3], Johannes Delgado-Ospina [1,4], María González-Locarno [3], Yarley Maza Pautt [3] and Clemencia Chaves-López [1]

[1] Faculty of Bioscience and Technology for Food, Agriculture and Environment, University of Teramo, Via R. Balzarini 1, 64100 Teramo, Italy; yyperaltaruiz@unite.it (Y.P.-R);jdelgado1@usbcali.edu.co (J.D.-O.); cchaveslopez@unite.it (C.C.-L.)

[2] Facultad de Ingeniería, Programa de Ingeniería Agroindustrial, Universidad del Atlántico, Carrera 30 Número 8–49, 081008, Puerto Colombia, Colombia; alsinning@mail.uniatlantico.edu.co

[3] Grupo de Investigación de fotoquímica y fotobiología, Universidad del Atlántico, Carrera 30 Número 8–49, 081008 Puerto, Colombia; mclaudiagonzalez@mail.uniatlantico.edu.co (M.G.-L); ymaza@mail.uniatlantico.edu.co (Y.M.P.)

[4] Grupo de Investigación Biotecnología, Facultad de Ingeniería, Universidad de San Buenaventura Cali, Carrera 122 # 6–65, 76001 Cali, Colombia; dpnavia@usbcali.edu.co

* Correspondence: carlosgrande@mail.uniatlantico.edu.co

Table S1. Volatile compounds identified in *Ruta graveolens* essential oil**.**

	Compound	RT	Amount Relative (%)	*LRI
Alcohol	2-undecanol	31.45	1.1	1304
	Manol	52.46	0.5	2076
	2-nonanol	23.84	3	1102
	1-nonanol	26.55	0.1	1172
Ketone	α-Thujone	24.25	0.1	1113
	2-undecanone	31.15	42.6	1296
	2-octanone	19.1	0.2	990
	2-decanone	27.38	4	1193
	(R)-(-)-Carvone	29.52	0.1	1251
	2-Dodecanone	34.93	2.9	1396
	2-nonanone	23.48	23.5	1094
	2-Tridecanone	38.44	2.5	1497
Ester	Octyl acetate	27.99	0.2	1209
	Benzyl acetate	46.24	1.7	1782
	1-Methylheptyl acetate	28.82	1.3	1232
	trans-farnesyl acetate	47.73	0.2	1834
	Benzyl 2-hydroxybenzoate	48.61	0.5	1887
Sesquiterpene	Nonyl acetate	31.62	0.7	1309
	Isodecanone	33.78	2.6	1366
	Geijerene	25.65	0.1	1149
	lsogeijerene C	29.98	0.1	1264
	Cogeijerene	30.36	0.2	1274
	Tetradecane	35.17	<0.1	1402
	Cis-β-Caryophyllene	35.7	0.1	1417
	Methyldecyl acetate	36.09	0.2	1429
	trans-β-Caryophyllene	36.28	0.8	1434
	(-)-Aromadendrene	36.53	0.9	1442
Sesquiterpene	Allo-aromadendrene	36.72	0.2	1447
	Isotridecanone	37.2	0.4	1461
	α -Humulene	37.53	1.1	1470
	γ -Muurolene	38.05	0.3	1485
	Geijerene	25.65	0.1	1149
	Valencene	38.64	0.2	1503
	α-Farnescene	38.75	0.2	1506
	γ-cadinene	39.31	0.2	1525
	σ-cadinene	39.41	0.5	1528
	α-Farnescene	43.44	0.2	1670
	(+)-cubenene	39.9	0.1	1545
Sesquiterpenoid	Viridiflorol	41.87	0.8	1611
	β-Eudesmol	43.52	0.2	1673
	Trans-Farnesol	44.72	0.3	1719
Furocoumarin	Ficusin	47.76	0.2	1849
	Chalepensin	54.8	1.1	2196
	N.I. (M+ 162)	29.76	0.9	1258
	N.I. (M+ 160)	43.61	0.3	1676
	N.I. (M+ 186)	43.7	1.1	1680
	N.I. (M+ 232)	47.25	1	1826
	N.I. (M+ 248)	51.94	0.4	2049
	N.I. (M+ 180)	52	0.1	2052

**Lineal Retention Index relative to C5–C24 n-alkanes on the. DB-5 column*

Table S2. Physical properties of the CS+RGEO coatings.

Essential Oil (%)	pH	Density (g/mL)	Viscosity Brookfield (cP)	Solids (%)	Particle Size (μm)
0	4.38 ± 0.01[a]	1.0017 ± 0.01[a]	106 ± 0.1[d]	2.56 ± 0.02[a]	N.D.
0.5	4.40 ± 0.01[b]	1.0076 ± 0.01[a]	74 ± 0.1[c]	3.71 ± 0.01[b]	1.00 ± 0.25[a]
1.0	4.41 ± 0.01[c]	1.0080 ± 0.01[a]	66 ± 0.1[b]	3.87 ± 0.02[c]	1.22 ± 0.32[a]
1.5	4.43 ± 0.01[d]	1.0088 ± 0.01[a]	28.5 ± 0.2[a]	3.59 ± 0.02[d]	1.57 ± 0.12[a]

[*] Values correspond to means ± standard deviation. Different superscript letters in the same column indicate significant differences between treatments ($p < 0.05$). N.D.= Not determined
